# Dual-Enhanced Pluronic Nanoformulated Methotrexate-Based Treatment Approach for Breast Cancer: Development and Evaluation of In Vitro and In Vivo Efficiency

**DOI:** 10.3390/pharmaceutics14122668

**Published:** 2022-11-30

**Authors:** Amira Mansour, Mohamed Y. Mahmoud, Alaa F. Bakr, Monira G. Ghoniem, Fatima A. Adam, Ibrahim M. El-Sherbiny

**Affiliations:** 1 Nanomedicine Research Labs, Center for Materials Science, Zewail City of Science & Technology, Giza 12578, Egypt; 2 Department of Toxicology and Forensic Medicine, Faculty of Veterinary Medicine, Cairo University, Giza 12211, Egypt; 3 Department of Pathology, Faculty of Veterinary Medicine, Cairo University, Giza 12211, Egypt; 4 Department of Chemistry, College of Science, Imam Mohammad Ibn Saud Islamic University (IMSIU), Riyadh 11623, Saudi Arabia

**Keywords:** methotrexate, piperine, bioenhancer, nanomicelles, pluronic, breast cancer

## Abstract

Breast cancer is a prevalent tumor and causes deadly metastatic complications. Myriad cancer types, including breast cancer, are effectively treated by methotrexate (MTX). However, MTX hydrophobicity, adverse effects and the development of resistance have inspired a search for new effective strategies to overcome these challenges. These may include the addition of a bioenhancer and/or encapsulation into appropriate nano-based carriers. In the present study, the anticancer effect of MTX was fortified through dual approaches. First, the concomitant use of piperine (PIP) as a bioenhancer with MTX, which was investigated in the MCF-7 cell line. The results depicted significantly lower IC_50_ values for the combination (PIP/MTX) than for MTX. Second, PIP and MTX were individually nanoformulated into F-127 pluronic nanomicelles (PIP-NMs) and F-127/P-105 mixed pluronic nanomicelles (MTX-MNMs), respectively, validated by several characterization techniques, and the re-investigated cytotoxicity of PIP-NMs and MTX-MNMs was fortified. Besides, the PIP-NMs/MTX-MNMs demonstrated further cytotoxicity enhancement. The PIP-NMs/MTX-MNMs combination was analyzed by flow cytometry to understand the cell death mechanism. Moreover, the in vivo assessment of PIP-NMs/MTX-MNMs was adopted through the Ehrlich ascites model, which revealed a significant reduction of the tumor weight. However, some results of the tumor markers showed that the addition of PIP-NMs to MTX-MNMs did not significantly enhance the antitumor effect.

## 1. Introduction

Breast cancer is the most observable cancer type affecting females in both developing and developed countries [1,2]. Globally, the reported recent incidence is 2.3 million cases; severely exceeding all other cancer types. Additionally, its mortality rate represents 6.9% from all cancer-related deaths [2]. Moreover, the metastatic complications, through the lymph nodes and though other organs, significantly contribute to increasing the death rate [3]. There are several pivotal treatment approaches for breast cancer, for example, mastectomy to remove the cancer tissue, while radiation and chemotherapy are additional approaches that can eradicate the cancer tissue and the metastatic cells [4]. Unfortunately, the morbidity and mortality rates are growing relentlessly despite the presence of several treatment strategies. These treatment failures are attributed to improper selection of drug(s), development of resistance, severe adverse effects and the high cost [4]. All these reasons represent the motivating factors to fulfill the treatment failures using a combination of drugs that may include bioenhancers or via specific delivery of the drugs with the aid of well-designed smart nano-drug delivery systems (DDSs).

Methotrexate (MTX) is an anticancer hydrophobic drug that exerts its cytotoxicity through the inhibition of dihydrofolate reductase (DHFR) enzyme that coverts the thymidylate synthase product; dihydrofolate to tetrahydrofolate. Consequently, this inhibition depletes the cells from thymidylate and prohibits the DNA and purine synthesis [5,6]. MTX has a wide range of biological anti-neoplastic activity, for instance, being active against colorectal cancer [7,8], leukemia [9], osteosarcoma [10,11] and remarkably, breast cancer [12]. Additionally, MTX is used to treat autoimmune diseases like rheumatoid arthritis [13]. Despite the reported potency of MTX, resistance has been developed due to its altered transport and accumulation intracellularly, as well as the prohibited drug-target binding [14]. Moreover, it has many systemic adverse effects, such as liver and kidney toxicity, bone marrow suppression and leukopenia, which all result from the off-target drug accumulation [15,16]. Additionally, MTX administration is hampered by its narrow therapeutic index, low selectivity, limited plasma life-time [15,17] and hydrophobicity [18]. In an attempt to enhance the efficiency and safety of MTX specifically for the treatment of breast cancer, it was used in combination with other anticancer agents, such as cyclophosphamide, fluorouracil, tamoxifen and epirubicin [19,20,21]. On the other hand, MTX was loaded into several types of nanoparticle (NPs) carriers, such as solid-lipid NPs [12], polymeric nanomicelles [22] or in association with other nanocarrier systems, such as magnetic NPs [23]. For example, MTX was loaded into polymeric nanomicelles based on a combination of two pluronic polymers; F127 and P105 to overcome the contaminant resistance by glycoprotein P (P-gp) efflux system [24].

Piperine (PIP) is a natural drug of piquant taste that is extracted mainly from black pepper (*Piper nigrum*) [25]. Among its medical applications are treatment of Alzheimer [26], anti-inflammatory [27], antihypertension [28] and anticancer effective against multiple types of cancer, including breast cancer [29]. Intriguingly, PIP demonstrated a pivotal role in enhancing the bioavailability of other drugs, which was attributed to its ability to inhibit P-gp and CYP3A4 enzyme [30,31]. Therefore, it is worthwhile that PIP has been incorporated with other anticancer agents as rapamycin [32], curcumin [33] and resveratrol [34] as a simultaneous bioenhancer to combat breast cancer. However, PIP is a hydrophobic drug and its absorption is limited by severe exposure to first pass metabolism [35]. To overcome these obstacles, some recent studies have reported the incorporation of PIP with other drugs into nanocarrier systems to enhance their bioavailability [36,37].

Pluronics^®^ are triblock copolymers consisting of variable chain lengths of polyethylene oxide (PEO) and polypropylene oxide (PPO). Due to their safety, biocompatibility, biodegradability and amphiphilic nature, they are used extensively to develop self-assembled nanoparticulate carriers as DDSs that enable the encapsulation of both hydrophilic and hydrophobic drugs [38,39,40,41]. Additionally, pluronics^®^ are able to escape uptake by macrophage cells because of their PEO hydrophilic shell. Besides, their tiny nano-size allows their accumulation within the cancer tissue through the enhanced permeability and retention (EPR) effect. Moreover, pluronics^®^ nanocarriers overcome drug resistance by the P-gp efflux system [42,43,44].

**Scheme 1:**
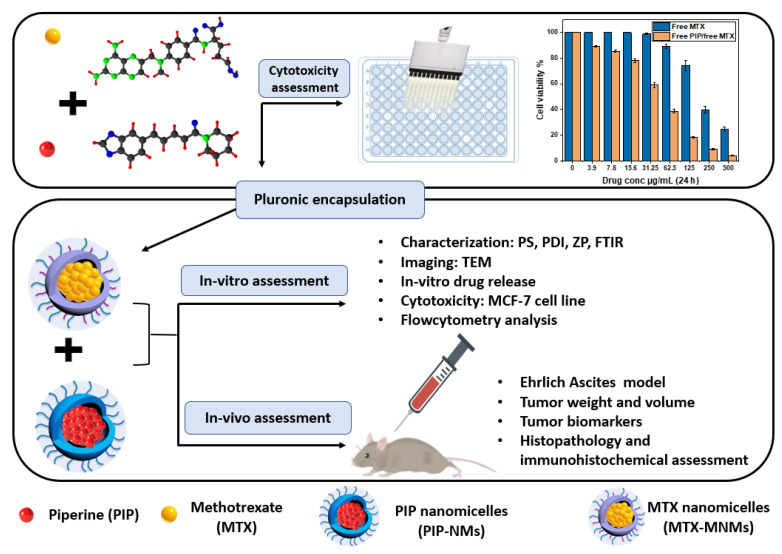
An illustration for the development of dual-enhanced methotrexate-based treatment approach for breast cancer.

Herein, the anticancer effect of MTX against breast cancer was fortified through two different strategies (Scheme 1). First, the simultaneous use of PIP as a bioenhancer of MTX’s anti-neoplastic activity, as per a certain ratio, followed by assessment of the cytotoxicity. Second, through the nanoformulation of PIP and MTX individually into pluronic nanomicelles that were validated by several characterization techniques. The cytotoxicity of nanoformulated individual drugs (PIP-NMS and MTX-MNMs) and their combination (PIP-MNs/MTX-MNMs), as per the previously used ratio, was reinvestigated. Besides, cell cycle analysis and apoptosis/necrosis evaluation of the combination was performed to understand the underpinned cell death mechanism. Additionally, the in vivo anti-neoplastic activity against Ehrlich ascites was tested using MTX-MNMs as positive control to investigate the anticancer effect of the combination; PIP-NMs/MTX-MNMs.

## 2. Materials and Methods

### 2.1. Materials

Pluronic^®^ F127, pluronic^®^ P105, MTX, triethylamine, methanol, acetone, dimethyl sulfoxide (DMSO), MTT and trypan blue were purchased from Sigma (St. Louis, MO, USA). Fetal bovine serum, DMEM, RPMI-1640, HEPES buffer solution, L-glutamine, gentamycin and 0.25% trypsin-EDTA were obtained from Lonza (Belgium). PIP (molecular weight of 285.34 Da and purity 98%) was provided by Alpha Aesar (Ward Hill, MA, USA).

### 2.2. Preparation of Plain Mixed Pluronic Nanomicelles (MNMs) and MTX-Loaded MNMs (MTX-NMs)

Plain (drug-free) mixed pluronic nanomicelles (MNMs) and MTX-loaded MNMs (MTX-MNMs) were prepared by thin-film hydration method [45]. Briefly, 300 mg of pluronics^®^ F127 and P105 (1:8 *w*/*w*) were dissolved in 5 mL methanol and 50 µL triethylamine. Then, a thin-film was formed with the aid of a rotary evaporator, followed with hydration by double volume of distilled water. The MTX-MNMs were prepared using the same procedure as the plain MNMs after adding a pre-determined amount (7 mg) of drug to the pluronics solution.

### 2.3. Preparation of Plain Pluronic Nanomicelles (NMs) and PIP-Loaded NMs (PIP-NMs)

Both plain NMs and PIP-loaded NMs (PIP-NMs) were prepared by nanoprecipitation method [46]. Either pluronic^®^ F127 only (40 mg) or mixed with a pre-determined amount (5 mg) of PIP were dissolved in 15 mL ethanol to develop plain NMs and drug-loaded NMs (PIP-NMs), respectively. Then, the ethanolic solution was added dropwise to a double volume of distilled water, followed by stirring overnight to evaporate the organic solvent [46].

### 2.4. Characterization

The particle size (PS), polydispersity index (PDI) and surface zeta potential (ZP) of plain MNMs, MTX-MNMs, plain pluronic F127 (NMs) and PIP-NMs were obtained by dynamic light scattering using a Zetasizer Nano ZS (Malvern Instruments, Malvern, UK) at room temperature. The morphology of all the developed NMs was visualized by HR-TEM (JEM-2100F; JEOL, USA). Lyophilized samples were investigated by Fourier transform infrared (FTIR) spectroscopy to obtain the main peaks for the chemical characterization using universal ATR module (Diamond/krs-5 crystal) by VERTEX 70 FTIR spectrometer 16 (Bruker Optics, Germany) in the range of 600–4000 cm^−1^.

The entrapment efficiency (EE%) and loading capacity (LC%) of the drugs were determined by the UV-Vis spectrophotometry (Evolution UV 600, Thermo Scientific) at 310 nm and 342 for MTX and PIP, respectively. The following equations were applied:

EE% = [(Drug_i_ − Drug_f_)/Drug_i_] × 100

LC% = [(Drug_i_ − Drug_f_)/(Drug_i_ + Pluronic)] × 100

where, Drug_i_ is the initial amount of drug, Drug_f_ is the amount of free (unentrapped) drug.

### 2.5. In Vitro Drug Release Study

The release profiles of free MTX drug and MTX from the developed MTX-MNMs were determined using the dialysis bag method (molecular weight cut-off 12,000) in PBS of different pH values; 7.4 and 5.5. Briefly, the samples were dispersed in pre-determined volume of PBS (as donor chamber) and left in a shaker incubator (110 rpm) at the physiological temperature 37 ± 1 °C. At certain time intervals, a fixed volume of aliquots was withdrawn and replaced with fresh buffer. The amount of the released drug was quantified using UV-Vis spectrophotometer at the corresponding wavelength (310 nm).

### 2.6. In Vitro Cytotoxicity Assay

The in vitro MTT cytotoxicity assay of free drugs (PIP and MTX), PIP-NMs and MTX-MNMs, in addition to the mixtures consisting of free PIP/free MTX and the PIP-NMs/MTX-MNMs, was performed using MCF-7 cells (human breast cancer cell line). The cells were obtained from the American Type Culture Collection (ATCC, Rockville, MD, USA). The cells were cultured on RPMI-1640 medium treated with 10% inactivated fetal calf serum and 50 µg/mL gentamycin and kept in 5% CO_2_ atmosphere for subculturing 2 to 3 times weekly. In a Corning^®^ 96-well tissue culture plate, the cells (5 × 10^4^ cell/well) were suspended and incubated for 1 day. Then, 12 serial concentrations of the tested formulation were added to the wells as triplicates. Six controls were used and were either the plain medium or 0.5% DMSO. After incubation for 1 day, the count of the viable cells was detected by MTT assay. Briefly, the media was replaced by 100 µL of RPMI-1640 without phenol red and the 10 µL of 12 mM solution were added to each well and incubated at 37 °C and 5% CO_2_ atmosphere for 4 h. Then, 85 µL of the media were discarded and replaced by 50 µL DMSO and incubated at 37 °C for 10 min. The number of the viable cells was determined by measuring the optical density at 590 nm using the equation:

[(ODt/ODc)] × 100%

ODt: the optical density of the cells treated with the formulations

ODc: the optical density of the untreated cells (control)

The IC_50_ was calculated from plotting the dose response versus the formulation concentration using the software Graphpad Prism (San Diego, CA, USA).

### 2.7. Apoptosis/Necrosis Assay by Flow Cytometry

In order to understand the mechanism of the cell death by the investigated mixture (PIP-NMs/MTX-MNMs), annexin V-FITC assay was carried out using FITC-annexin V (Becton Dickinson BD Pharmingen^TM^, Heidelberg, Germany) using the IC_50_/24 h. Briefly, the MCF-7 cells were cultured and incubated with the formulation for 1 day while the negative control cells were incubated with the plain medium. Afterwards, the cells were collected and washed with PBS twice. The cells were then washed by the binding buffer (100 µL) and FTIC-annexin V (1 µL) and incubated at 4 °C for 40 min. The cells were rinsed and resuspended in the binding buffer (150 µL) and DAPI (1 µL/mL in PBS) (Invitrogen, Life Technologies, Darmstadt, Germany). Finally, the cells were tested by a flow cytometer (BD FACS Calibur, BD Biosciences, San Jose, CA, USA).

### 2.8. Cell Cycle Analysis

The effect of the mixture (PIP-NMs/MTX-MNMs) on cell cycle distribution was determined using MCF-7 cells by the flow cytometry. CycleTEST^TM^ PLUS DNA Reagent Kit (Becton Dickinson Immunocytometry Systems, San Jose, CA, USA) was used to investigate the mixture-treated and the control (untreated) cells followed by staining by propodium iodide then analyzed by the cytometer. Then, CellQuest software (Becton Dickinson Immunocytometry Systems, San Jose, CA, USA) was used to calculate the cell cycle distribution.

### 2.9. In Vivo Assessment

From the animal house of VACSERA, Egypt, 30 Balb/c mice were used to develop this test. The mice weight was in the range of 20 to 40 g each. The in vivo experiment was performed after receiving the approval from the research ethics committee for experimental and clinical studies at the Faculty of Pharmacy, Ain Shams University, Cairo, Egypt that complies with the United Kingdom Animals Scientific Procedures Act, 1986, and the European Union directive 2010/63/EU for animal experiments, as well as the ARRIVE guidelines. The Ehrlich tumor was induced in the mice by intramuscular injection in the right thigh of viable tumor suspension (1.25 × 10^7^ cells/mL) obtained from a female mice ascitic fluid. The mice were left for 2 weeks until a sensible solid tumor was obtained, with a size of more than 100 mm^3^. The mice were injected intraperitoneally on a daily basis for 3 weeks and divided into 3 groups:

Group 1: negative control (received distilled water).

Group 2: mice received MTX-MNMs (a dose equivalent to 6 mg/kg MTX) [12].

Group 3: mice received PIP-NMs/MTX-MNMs (a dose equivalent to 0.1 mg/kg PIP and 6 mg/kg MTX, respectively) [12,47].

#### 2.9.1. Tumor Weight and Volume Monitoring

The tumor volume was monitored during the experiment at different periods (0, 7, 14 and 21 days) by vernier caliper (Mitutoyo Corporation, Kawasaki, Japan) and calculated according to the equation:

$$\mathrm{Tumor}\;\mathrm{volume}\;({\mathrm{mm}}^{3})=4\pi \;{\left(\;\frac{A}{2}\;\right)}^{2}\times \left(\;\frac{B}{2}\;\right)$$

*A*: minor tumor axis, *B*: major tumor axis

However, the tumor weight was detected after the animals’ sacrification.

#### 2.9.2. Tumor Growth Biomarkers Measurements

After the animals’ euthanasia, the tumor tissue was obtained and cleaned with ice. Then, by polytron homogenizer, the tumor was homogenized and diluted by 0.05 M PBS (pH 7.4) to represent 10% of the solution. Afterwards, it was preserved as aliquots in −80 °C for further analyses to detect cyclin D1, p-38 MAPK (mitogen-activated protein kinase), apoptosis, AKT, malondialdehyde (MDA) and superoxide dismutase (SOD). Cyclin D1 and p-38 MAPK were detected using ELISA kit (Abbexa^®^, Houston, TX, USA) and Total SimpleStep ELISA^®^ Kit, respectively as per the manufacturer’s instructions. DNA fragments were quantified as representative for the apoptotic cells using Apo-BrdU In Situ DNA Fragmentation Assay Kit (BioVision^®^, Milpitas, CA, USA) as per the manufacturer’s instructions. The AKT level was investigated using BCA test. Briefly, the tumor tissue was fragmented in lysis buffer by a homogenizer followed by centrifugation to isolate the supernatant. To allow cells lysis, sodium dodecyl sulfate (SDS) was added and the solution was then transferred to SDS-PAGE electrophoresis loaded with molecular weight marker. Then, the isolated proteins were transferred to a nitrocellulose membrane loaded with AKT antibodies (9272, Cell signaling^®^, Danvers, MA, USA) and left for 1 h. Then, the membrane was rinsed and incubated with horseradish peroxidase-conjugated secondary antibodies for 45 min. Finally, the samples were quantified by western blot through enhanced chemiluminescence (ECL). In an attempt to estimate the oxidative stress, MDA and SOD were detected by the Lipid Peroxidation Colorimetric/Fluorometric Assay Kit (BioVision^®^, Milpitas, CA, USA) and Biodiagnostic, Egypt, respectively, followed by colorimetric and absorbance measurement at 532 and 405 nm, respectively.

#### 2.9.3. Histopathological and Immunohistopathological Assessment

After euthanasia, the tumor and the surrounding muscle were dissected and fixed in a neutral buffered formaldehyde (10%) for 24 h. Later, the tissues were dehydrated, set in paraffin blocks, and sliced at a thickness of 5 μm, transversely. Subsequently, the slides were stained with hematoxylin and eosin (H&E) and observed using Olympus BX43 light microscope linked with a digital camera (DP27) and CellSens dimensions software. The histological damage score was assessed at 200×, as previously described [48].

For immunohistochemistry analysis, tissue samples were mounted on positive glass slides, deparaffinized, rehydrated and incubated with the primary mouse monoclonal antibody of TGF-β and Bcl-2 (1:100) according to the manufacturer’s instructions (Santa Cruz). ImageJ software was used to estimate the semi-quantitative of the area % of positive results (6 samples/group/200×).

## 3. Results

### 3.1. Preparation and Characterization of Plain and Drugs-Loaded Pluronic Nanomicelles

The preparation of the plain pluronic nanomicelles (NMs) and PIP-NMs was attempted by the nanoprecipitation method [46]. On the other hand, plain mixed nanomicelles (MNMs) and MTX-MNMs were prepared by the thin film hydration method [45]. The average PS, PDI and ZP are summarized in Table 1. The plain MNMs depicted PS of 26.3 nm while when loaded with the drug (MTX), the MTX-MNMs showed an increase of the PS to 104.6 nm. The PS of the plain NMs was 96.3, while the PIP-loaded NMs (PIP-NMs) attained a PS value of 114.2 nm. The PDI values for all the prepared plain and drug(s)-loaded NMs ranged from 0.19 to 0.43 which indicate a narrow PS distribution. As can also be noted from Table 1, the plain NMs exhibited a surface charge (ZP) of −19.4 mV, while after the PIP loading, the ZP changed to −8.02 mV. On the other hand, the ZP of the plain MNMs was −4.42 mV and changed to −3.47 mV after MTX loading.

**Figure 1:**
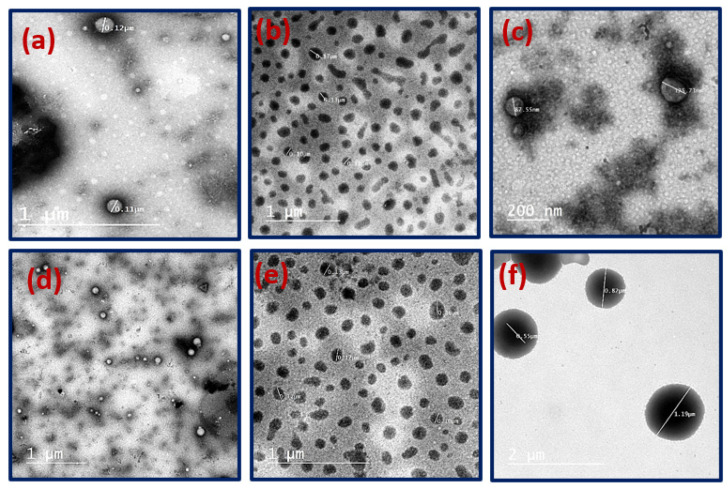
TEM micrographs of (**a**) plain pluronic nanomicelles (NMs), (**b**,**c**) PIP-loaded NMs (PIP-NMs) at different magnifications, (**d**) plain mixed pluronic nanomicelles (MNMs), and (**e**,**f**) MTX-loaded MNMs (MTX-MNMs) at different magnifications.

The morphology of the developed plain and drugs-loaded nanomicelles was investigated by TEM (Figure 1). As can be noted from the figure, all the plain and drug (PIP or MTX)-loaded NMs were spherical in shape. The average sizes of the plain NMs (Figure 1a) and PIP-NMs (Figure 1b,c) were about 78 and 110 nm, respectively, while the sizes of the plain MNMs (Figure 1d) and the MTX-MNMs (Figure 1e,f) were around 27 and 80 nm, respectively.

The EE% and LC% of each of the two drugs; PIP and MTX loaded into the developed PIP-NMs and MTX-MNMs, were estimated by UV-Vis measurement. In the case of the PIP-loaded NMs, the EE% and LC% were found to be 82% and 6%, respectively. While in the case of the MTX-MNMs, the EE% and LC% were determined to be 85% and 2.5%, respectively. These results are in consistency with the literature [24,46].

**Figure 2:**
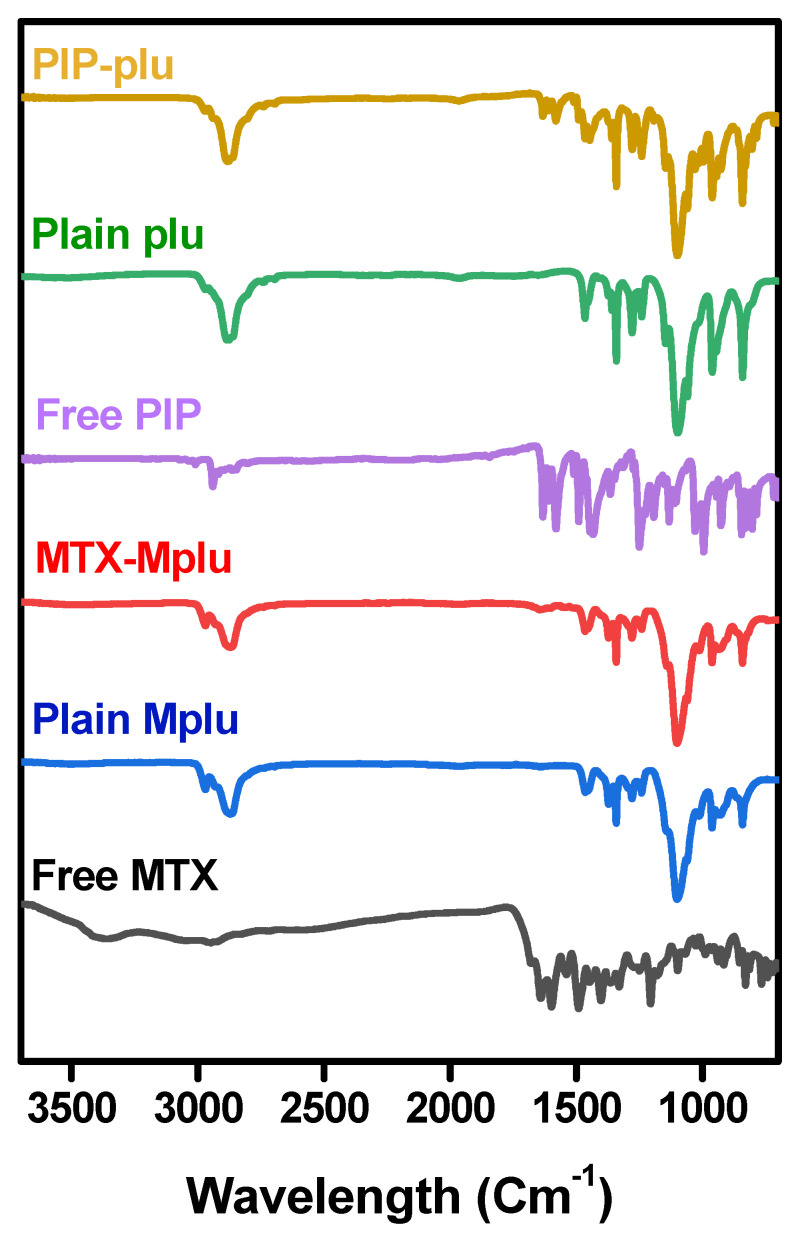
FTIR spectrum of free MTX, plain mixed pluronic nanomicelles (MNMs), MTX-MNMs free PIP, plain pluronic nanomicelles (NMs) and PIP-NMs.

Free drugs (PIP and MTX), plain nanomicelles (NMs and MNMs) and the drugs-loaded NMs (PIP-NMs and MTX-MNMs) were scanned by FTIR to confirm the nanomicelles formation in addition to the physical drugs’ encapsulation, as shown in Figure 2. The free PIP spectrum showed characteristic peaks of symmetric and asymmetric stretching of aliphatic C-H at 2871 and 2942 cm^−1^, respectively, -CO-N stretching at 1634 cm^−1^, symmetric and asymmetric stretching of C=C diene at 1612–1584 cm^−1^, respectively, aromatic stretching of C=C at 1518 cm^−1^, CH_2_ bending at 1428 cm^−1^, asymmetric and symmetric stretching of =C-O-C at 1254–1233 and 1045 cm^−1^, respectively, C-O stretching at 958 cm^−1^ and C-H bending at 847–802 cm^−1^. The plain pluronic NMs and MNMs spectra showed C-H stretching vibration peak at 2890 cm^−1^, O-H bending at 1342 cm^−1^ and C-O-C stretching vibration at 1110 cm^−1^ [46]. From the FTIR spectra, the NMs formation and the PIP incorporation into the NMs was confirmed by the presence of their characteristic peaks in the PIP-loaded NMs (PIP-NMs) spectrum. The free MTX IR spectrum showed the characteristic broad peak at 3417 cm^−1^ corresponding to the stretching of O-H of the carboxyl group, 2957 cm^−1^ of stretching of primary amine group (N-H), 1603–1647 cm^−1^ from C=O stretching from both the amide and carboxyl groups, 1494–1519 cm^−1^ from bending of N-H group, 1425–1229 cm^−1^ stretching of C-O of the carboxyl group, 885 cm^−1^ from O-H bending and 828 cm^−1^ from hydrogens on the aromatic ring [49]. From the MTX-loaded MNMs (MTX-MNMs) spectrum, these peaks were not obvious, and the spectrum of MTX-MNMs was similar to that of the plain MNMs, which emphasize the successful physical incorporation of the MTX inside the micelles.

### 3.2. In Vitro Drug Release

**Figure 3:**
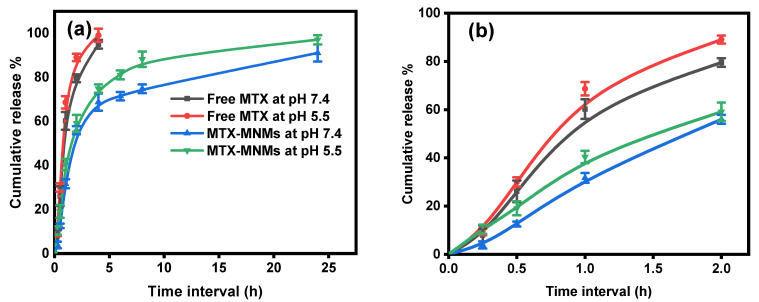
In vitro release profiles of the free MTX and MTX-loaded MNMs (MTX-MNMs) at two different pH values (7.4 and 5.4) for 24 h (**a**) and at the first 4 h (**b**).

The release profiles of the free MTX and MTX-loaded MNMs (MTX-MNMs) at two different pH values (7.4 and 5.4) representing the physiological and cancer tissue pHs, respectively, are shown in Figure 3. As can be noted from the figure, the free drug, MTX, conferred a fast burst release at both investigated pH values, as more than 90% of the drug was released within the first 4 h. However, in the case of the MTX-MNMs, the drug showed a fast initial release of about 60% at both pH values within the first 3 h, followed by a sustained release pattern. Additionally, as can be depicted from the figure, the nanoformulated MTX showed a relatively higher release values at pH 5.4 than that attained at pH 7.4. For instance, about 90% of MTX was released in the acidic pH (5.4) as compared to 70% of drug released at pH 7.4 within 8 h. The remaining MTX amount was released at a slower rate to be almost completed after 24 h.

### 3.3. In Vitro Cytotoxicity

The cytotoxicity assessment was performed using the MCF-7 cell line by MTT test. First, the IC_50_ values of the free PIP at 24 h and of free MTX at 24 and 48 h were determined using serial concentrations ranging from 0 to 500 µg/mL. The free PIP IC_50_ at 24 h was found to be 183 μg/mL while the free MTX IC_50_ values at 24 and 48 h were found to be 213 μg/mL and 115.3 μg/mL, respectively (Table 2).

**Figure 4:**
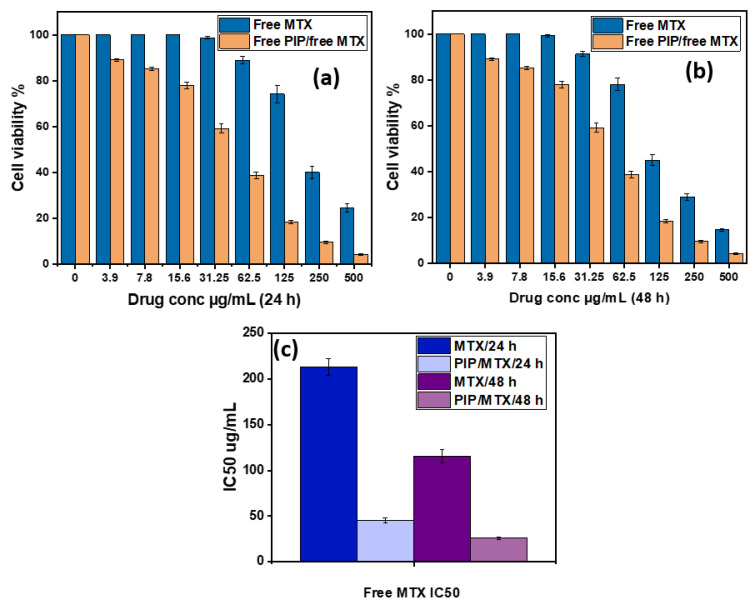
The cytotoxicity test on MCF-7 cells of free MTX individually, and the mixture of free drugs (PIP/MTX) using half IC_50_ of the bioenhancer, free PIP at (**a**) 24 and (**b**) 48 h, and (**c**) the IC_50_ values from the cytotoxicity assay of free MTX only and of the mixture of free drugs (PIP/MTX) at 24 and 48 h, respectively.

Then, to assess the bioenhancing effect of the investigated bioenhancer (PIP) on the cytotoxicity of MTX, the IC_50_ values at 24 and 48 h of the free MTX were re-determined after its mixing with half of the previously determined IC_50_ concentration of the bioenhancer (PIP). From the results, the IC_50_ values of the free MTX decreased significantly at both investigated time intervals (24 and 48 h) to be 45.23 μg/mL (*p* < 0.0001) and 25.84 μg/mL (*p* < 0.0001) at 24 and 48 h, respectively when compared to the MTX alone (Figure 4).

**Figure 5:**
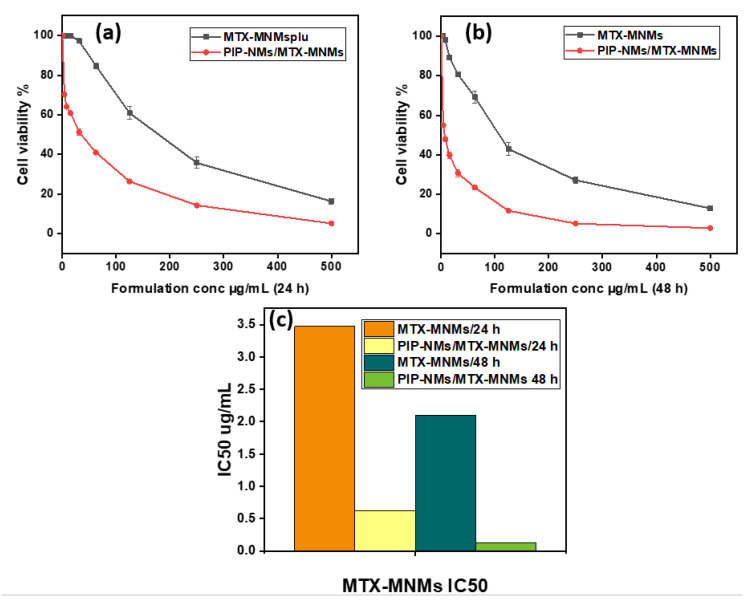
(**a**,**b**) The cytotoxicity test on MCF-7 cells at 24 h (**a**) and 48 h (**b**) of MTX-MNMs individually and the mixture of PIP-NMs/MTX-MNMs using half PIP-NMs IC_50_, and (**c**) the IC_50_ values from the cytotoxicity assay of MTX-MNMs only and the mixture of PIP-NMs/MTX-MNMs at 24 and 48 h, respectively.

On the other hand, the IC_50_ values of the PIP-NMs at 24 h and MTX-MNMs at 24 and 48 h were determined (Figure 5). The encapsulation of both PIP and MTX into pluronic nanocarriers was found to enhance the cytotoxicity (and consequently attain lower IC_50_ values) when compared to the free drugs counterparts. The IC_50_ of the PIP-NMs was decreased from 183 μg/mL to be 11.84 μg/mL (*p* < 0.0001). Additionally, the IC_50_ of the MTX-MNMs significantly decreased when compared to the free form of the drug at both time intervals; 24 and 48 h to be 3.47 μg/mL (*p* < 0.0001) and 2.099 μg/mL (*p* < 0.0001), respectively (Table 2).

Due to the significant difference in the cytotoxic effect of both drugs; PIP and MTX after encapsulation into pluronic NMs, and to assess the bioenhancing effect of the investigated nanoformulated bioenhancer (PIP-NMs) on the cytotoxicity of MTX-MNMs, the IC_50_ values at 24 and 48 h of the MTX-MNMs were re-determined after its mixing with half of the previously determined IC_50_ concentration of the nanobioenhancer (PIP-NMs), as shown in Figure 5a,b and Table 2. The cytotoxicity of the MTX-MNMs was enhanced as the IC_50_ at 24 h was decreased about 5-fold to be 0.627 µg/mL (*p* < 0.0006). Additionally, the IC_50_ at 48 h revealed a much potent cytotoxic effect by decreasing the IC_50_ about 16-fold to be 0.128 µg/mL (*p* < 0.0001) (Figure 5c).

### 3.4. Apoptosis/Necrosis Assay and Cell Cycle Analysis by Flow Cytometry

**Figure 6:**
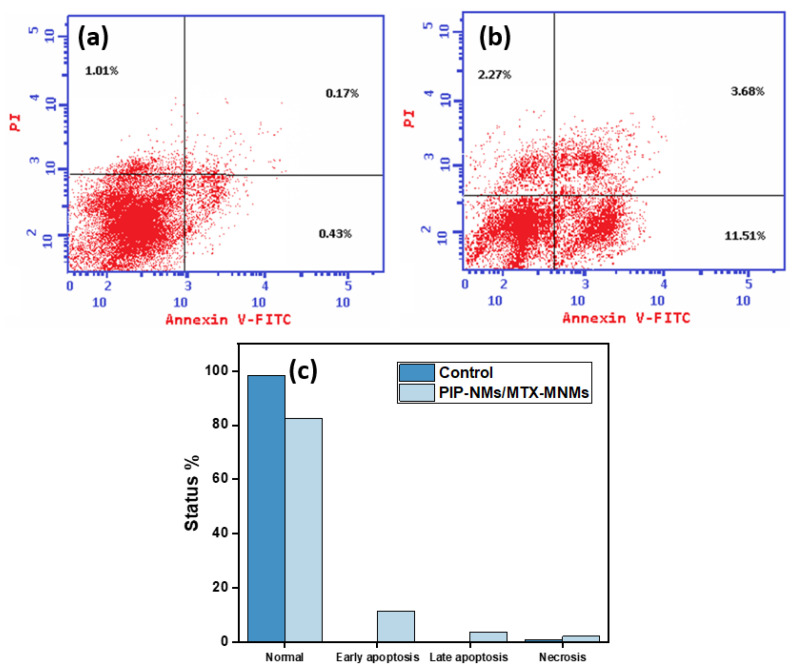
(**a**,**b**) Cytograms illustrating the induced cell death in (**a**) untreated MCF-7 cells as a control and (**b**) MCF-7 cells treated with PIP-NMs/MTX-MNMs using the IC_50_ obtained at 24 h, and (**c**) the cell death percent attained at the early apoptosis, late apoptosis and necrosis of the PIP-NMs/MTX-MNMs combination as compared to the untreated MCF-7 control cells.

To understand the underpinned mechanism by which the mixture, PIP-NMs/MTX-MNMs, induces cell death, flowcytometric analysis was performed using the MCF-7 cells. The annexin-V/propidium iodide test was performed to distinguish the apoptotic from necrotic cells (Figure 6). Both the early and late apoptosis were enhanced to a great extent as compared to the control cells. For instance, the early apoptosis was increased significantly (*p* < 0.0001), from 0.43% to 11.51%, and the late apoptosis increased significantly (*p* < 0.0001), from 0.17% to 3.68%. Besides, the necrotic cells were almost doubled from 1.01% to 2.27% (*p* < 0.0001). Based on the obtained results, the investigated PIP-NMs/MTX-MNMs combination was found to induce cell death through mainly enhancing both the early and late apoptotic stages (Figure 6c).

**Figure 7:**
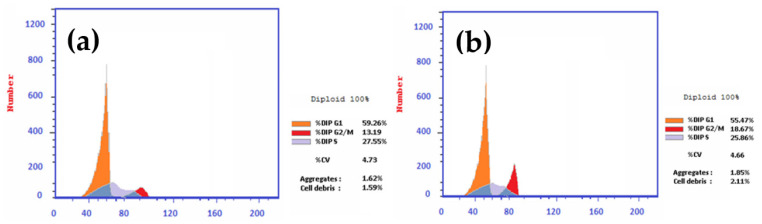
Cell cycle distribution histograms of (**a**) un-treated MCF-7 cells as control and (**b**) cells incubated with the mixture PIP-NMs/MTX-MNMs using IC_50_ at 24 h.

Cell cycle analysis was performed to investigate the cell cycle kinetics induced by PIP-NMs/MTX-MNMs using the IC_50_ at 24 h as compared to the untreated MCF-7 cells as negative control (Figure 7). At the G0/G1, the investigated PIP-NMs/MTX-MNMs combination decreased the cells population from 59.26% to 55.47%, while the population decrease attained in the S phase was of a lower magnitude (from 27.55% to 25.86%) with *p* = 0.0027 and 0.0417, respectively. In the G2/M phase, the PIP-NMs/MTX-MNMs significantly (*p* < 0.0001) increased the cells arrest by about 1.5 times (from 13.19 to 18.67%). Thus, the cell cycle arrest occurs mainly in the G2/M phase, followed by the G0/G1 phase.

### 3.5. In Vivo Assessment

#### 3.5.1. Tumor Volume and Weight Reduction Measurements

**Figure 8:**
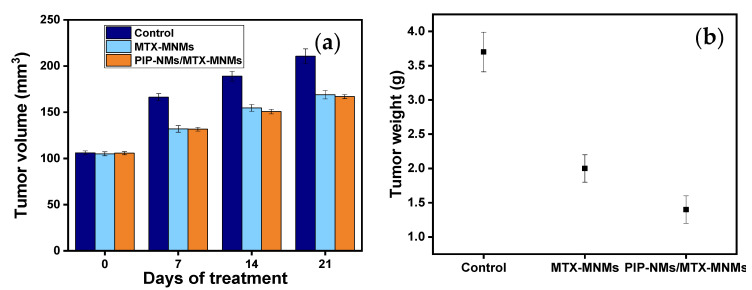
(**a**) Tumor volume (mm^3^) and (**b**) tumor weight of mice treated with distilled water as a control group, MTX-MNMs and PIP-NMs/MTX-MNMs combination. Data represented as mean ± SD (*n* = 7).

The mixture of the nanoformulated drugs was tested to evaluate any additional effect of the concomitant use of the bioenhancer (PIP)-loaded NMs (PIP-NMs) along with the MTX-MNMs as per the same ratio of the in vitro assessment. The tumor volume was determined at fixed days in all the mice groups, including the control group (Figure 8a). The tumor volume decreased significantly in the groups that received either the MTX-MNMs or the PIP-NMs/MTX-MNMs drug nano-combination when compared to the control group (*p* < 0.05). On the other hand, there was no observable significant difference in the tumor volume between the groups that received MTX-MMNs and PIP-NMs/MTX-MNMs. Tumor weight was also determined at the end of the treatment duration, as per Figure 8b. As can be depicted from the figure, the tumor weight of the nanoformulations-treated groups was substantially lower than that of the control (*p* < 0.05), with the PIP-NMs/MTX-MNMs-treated group exerting the most tumor weight reduction (*p* < 0.005) when compared to the MTX/MNMs-treated group.

#### 3.5.2. Tumor Growth Biomarkers Measurements

##### Cyclin D1 Concentration Assessment

Upon investigation of the cyclin D1 concentration, a significant reduction in its level was noted in both nanoformulations (MTX-MNMs and PIP-NMs/MTX-MNMs) when compared to the control group (*p* < 0.005), Table 3. Consequently, both the nanoformulations are inhibiting the cell cycle by downregulating the cyclin D1 level. However, it is worth mentioning that addition of PIP-NMs to the MTX-MNMs did not further decrease the cyclin D1 level.

##### p-38 MAPK Levels Assessment

The concentration of p-38 MAPK was determined in both the investigated nanoformulations-treated groups as compared to the control one. From the results, it can be elicited that there is a significant decrease of the p-38 MAPK level in both nanoformulations-treated groups (*p* < 0.005), Table 3. Besides, it is noticeable that the PIP-NMs/MTX-MNMs combination demonstrated a lowered p-38 MAPK level relative to the MTX-MNMs (*p* < 0.05).

##### Oxidative Stress Assessment

**Figure 9:**
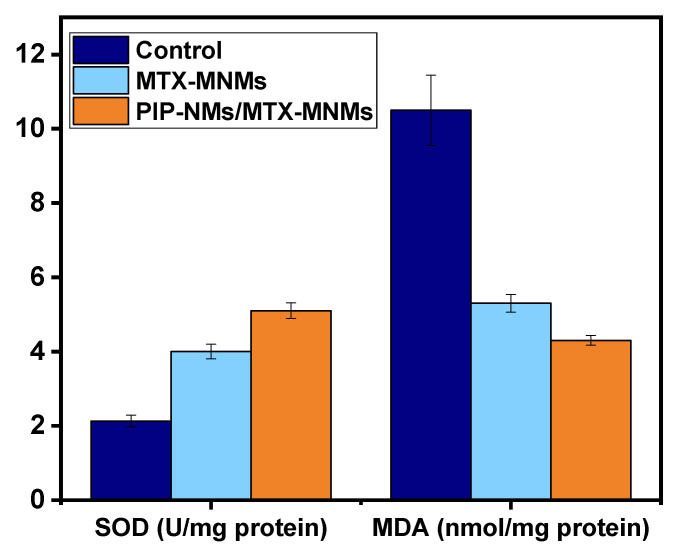
Superoxide dismutase (SOD) levels (U/mg protein) and malondialdehyde levels (nmol/mg protein) in mice treated with MTX-MNMs and PIP-NMs/MTX-MNMs-treated groups relative to the control mice. Data represented as mean ± SD (*n* = 7).

As an attempt to assess the oxidative stress, SOD and MDA concentrations were detected (Figure 9). The results demonstrated a significant increase of the SOD in both MTX-MNMs and PIP-NMs/MTX-MNMs-treated groups as compared to the control group (*p* < 0.05). Additionally, the addition of the PIP-NMs has enhanced the SOD concentration compared to the MTX-MNMs-treated group (*p* < 0.05). Consequently, the SOD elevated level upon treatment with the nanoformulation (MTX-MNMs) and the further elevation attained in the PIP-NMs/MTX-MNMs-treated group indicates the high number of the existing free radicals. These results are in concordance with the previously reported relation between MTX administration and the increase of SOD level [50].

Upon the assessment of the MDA level depicted in Figure 9, the control group displayed a significantly elevated level of MDA as compared to the nanoformulation-treated groups, which exhibited a significant reduction in MDA level (*p* < 0.001). Besides, addition of the nanoformulated bioenhancer (PIP-NMs) to the MTX-MNMs further reduced the MDA level, though this further reduction was not statistically significant (*p* ≥ 0.05).

##### AKT Assessment and Tunnel Assay

**Figure 10:**
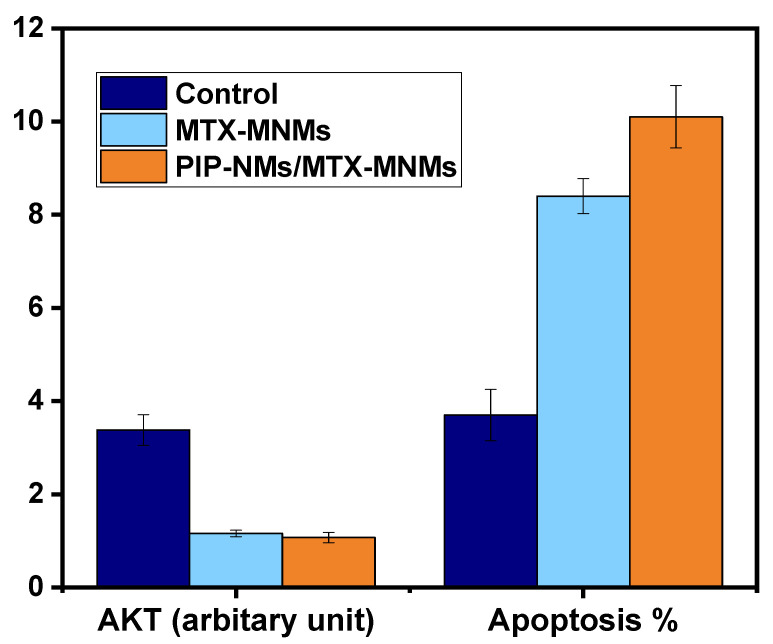
AKT (arbitrary unit) and apoptosis (%) determined in the MTX-MNMs and PIP-NMs/MTX-MNMs-treated groups relative to the control group. Data represented as mean ± SD (*n* = 7).

The induced apoptosis was determined through phosphorylated AKT (pAKT) and tunnel assay determination by western blot (Figure 10). The obtained results indicated that the AKT level was almost diminished in the nanoformulations-treated groups when compared to the control group (*p* < 0.005). However, the AKT level was almost the same in both the MTX-MNMs and PIP-NMs/MTX-MNMs-treated groups. As an additional attempt to investigate the apoptosis, the DNA fragmentation was assessed as apoptosis biomarker in the mammalian cells and considered as an indication of the anticancer treatment efficiency. From the results depicted in Figure 10, the PIP-NMs/MTX-MNMs-treated group exerted the highest apoptotic activity when compared to that of the MTX-MNMs-treated group (*p* < 0.05) and the control group (*p* < 0.005).

#### 3.5.3. Histopathology and Immunohistochemical Assessment

**Figure 11:**
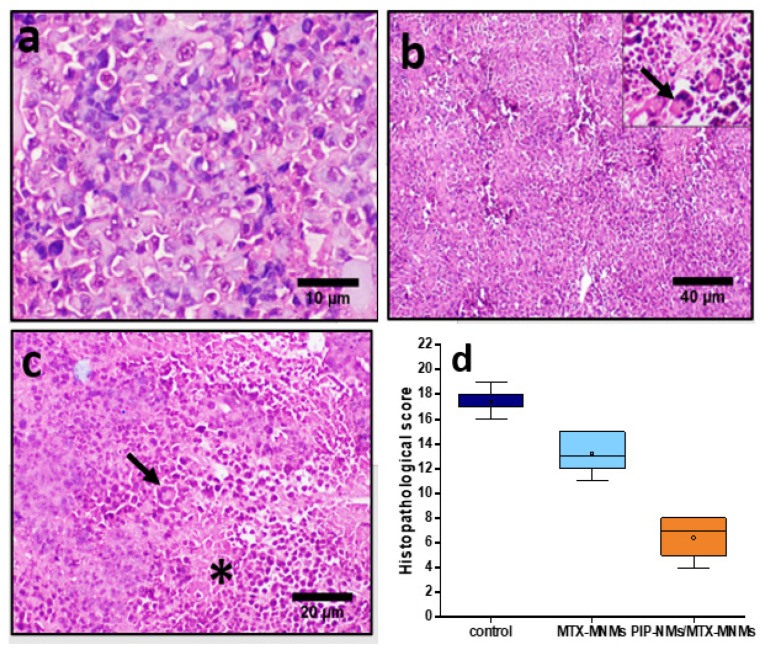
Assessment of muscular tissue inoculated with Ehrlich ascites tumor cells and stained with H&E of the (**a**) control group, (**b**) MTX-MNMs-treated group, (**c**) PIP-NMs/MTX-MNMs-treated group and (**d**) the area (%) analysis of the positive reaction. Symbols on the figure are revealed as follows: black arrow (giant cells) and star (coagulative necrosis).

The histological examination and observation of all groups are shown in Figure 11. The control group samples revealed a significant infiltration of active-darkly staining Ehrlich tumor cells with a high grade of malignancy features, including atypical mitosis and pleomorphism. No histological improvement was noticed in the MTX-MNMs-treated group. A significant regression in the infiltration of tumor mass between muscle fibers, as well as cells count, was recorded in the PIP-NMs/MTX-MNMs-treated group relative to the control group (*p* ≤ 0.01).

**Figure 12:**
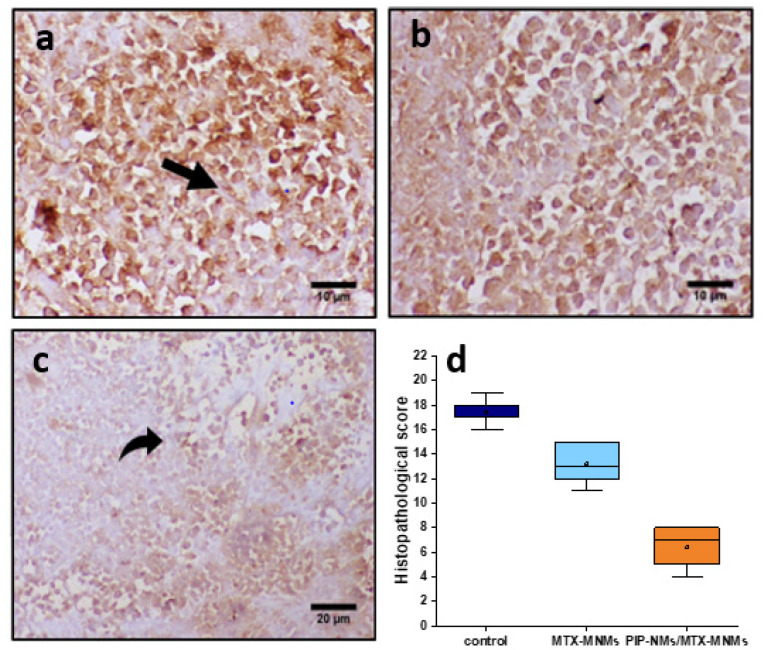
Immunohistochemical staining of TGF-β for (**a**) the control group, (**b**) MTX-MNMs-treated group, (**c**) PIP-NMs/MTX-MNMs-treated group, and (**d**) the area (%) of TGF-β. Straight and curved arrows are pointing to positive expression, and weak expression, respectively.

**Figure 13:**
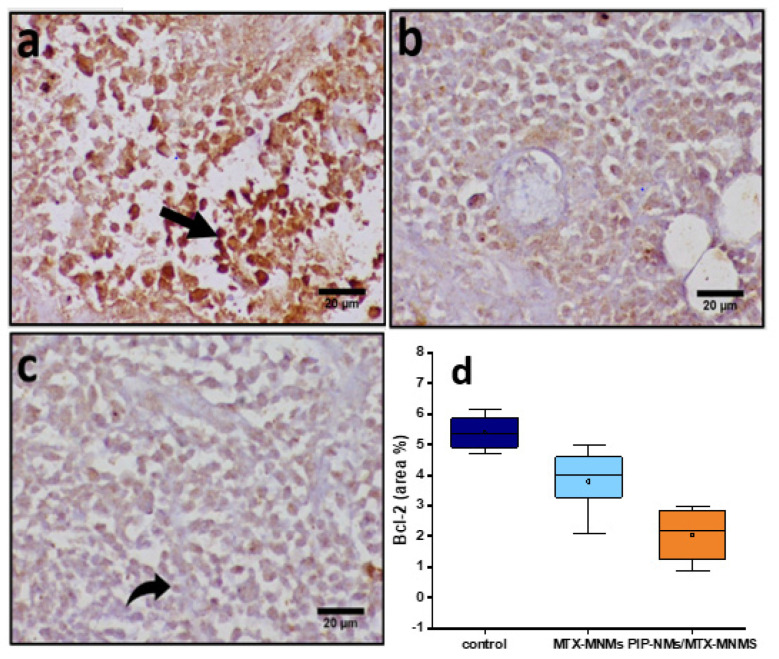
Immunohistochemical staining of Bcl-2 for (**a**) the control group, (**b**) MTX-MNMs-treated group, (**c**) PIP-NMs/MTX-MNMs-treated group, and (**d**) the area (%) of Bcl-2. Straight and curved arrows point to positive expression, and weak expression, respectively.

Concerning immunohistochemical analysis, a strong positive expression of TGF-β and Bcl-2 that appeared as brown coloration of Ehrlich cells was observed in control group. Treatment with MTX-MNMs did not significantly reduce the expression of TGF-β (Figure 12) and Bcl-2 (Figure 13) as the cells appeared almost similar to the control group. However, the treatment with PIP-NMs/MTX-MNMs significantly downregulated their expression.

## 4. Discussion

The aim of the study is to enhance the anticancer effect of MTX against breast cancer and evalute that in vitro and in vivo using the MCF-7 cell line and the Ehrlich ascites model, respectively. The MCF-7 cell line was used in this study, as it is the most common human breast cancer representative and the most studied in research [51]. This enhancement was achieved through two approaches; the first one included the simultaneous use of PIP as a bioenhancer for the anticancer effect of MTX. The second approach involved the encapsulation of each drug individually inside pluronic NMs, followed by mixing the pluronic-loaded drugs to examine the overall cytotoxicity. The nanomicellization and physical drug loading were confirmed with the aid of a diversity of physicochemical characterization techniques as dynamic light scattering, zeta potential, TEM images and FTIR.

Due to the hydrophobic nature of both drugs, PIP and MTX, their therapeutic bioavailability is limited. Therefore, they were encapsulated individually inside pluronic NMs. PIP was successfully encapsulated into pluronic^@^ F127 NMs using a nanoprecipitation method with an EE% of 82%. In the case of MTX, it was loaded successfully (with EE% 85%) into mixed NMs (MNMs) of both pluronic^@^ F127 and P105 using a thin-film hydration method. MTX incorporation into MNMs was to avoid the large particle size obtained upon its encapsulation into pluronic^@^ F127 alone [52]. Pluronic^@^ F127 and P105 are amphiphilic polymers and their hydrophobic parts are very close in length; F127 PPO: 64 and P015 PPO: 56. Therefore, upon the preparation of the MNMs from these two pluronic polymers, they exhibited cooperative aggregation [52], which facilitated the physical encapsulation of MTX. Both of the used preparation methods (nanoprecipitation and thin film hydration) were previously reported for the fabrication of NMs and were shown to be suitable for the encapsulation of hydrophobic drugs, so these results are in concordance with the literature [24,38,39,40,46]. The obtained high EE% values may be attributed to the hydrophobic part of the pluronic carriers forming the inner core of the micelles (NMs and MNMs), which is stabilized by escaping the hydrophilic medium. On the other hand, the hydrophilic parts of pluronic carriers stabilize the resulting nanoformulation by being exposed to the surrounding aqueous medium [53].

The release study was performed to identify the release rate of free MTX and the MTX from MTX-MNMs at two different pH values (7.4 and 5.4), representing the physiological pH and the cancer tissue pH, respectively, while maintaining the sink conditions. Free MTX exhibited a fast release rate, as almost all the drug was released after 4 h. However, the MTX-MNMs showed an initial burst release followed by a slower release pattern, with an observable faster rate at the acidic pH. In both pH values, the release was relatively fast (within 24 h) due to the physical entrapment of the drug, which facilitates its liberation from the micelles [24]. Additionally, the solvent effect on the micelles, and therefore, micelles erosion and disruption, aid the release of the drug. The faster release of MTX from MTX-MNMs at the acidic pH may be attributed to the enhanced interaction between the H^+^ and the PEO of the pluronics. This interaction can lead to a decreased chain packing, and thus, loosen micelles structure due to charge repulsion [54].

The bioenhancing effect of PIP on MTX anticancer activity was investigated by using both drugs in their free forms. PIP was added to MTX as per certain ratio (half of PIP IC_50_) so that the PIP amount did not affect more than 25% of the cells’ population [55]. Consequently, to determine this amount, the IC_50_ values of PIP and MTX were first detected individually. Afterwards, the IC_50_ value of the PIP/MTX mixture was reinvestigated after the addition of the half PIP IC_50_ to MTX, as previously mentioned. From the results, the addition of PIP enhanced the bioavailability and fortified the anticancer effect of free MTX. This was evidenced by the decrease of the IC_50_ value of MTX at 24 and 48 h after PIP addition as compared to the MTX alone. Consequently, the mixing of PIP with MTX as free drugs enhanced the cytotoxicity against MCF-7 cells.

The IC_50_ values of PIP and MTX were also tested individually after their encapsulation into pluronic nanocarriers (PIP-NMs and MTX-MNMs). This nanoencapsulation was found to enhance the cytotoxic effect of both drugs due to the increased solubilization and thus, the better bioavailability. Besides, these individual nanoformulated drugs demonstrated lower IC_50_ values as compared to their free drugs counterparts, which may be attributed to the role played by the pluronic nanocarrier in enhancing the anticancer activity, where pluronics are able to escape the uptake by macrophage cells because of their PEO hydrophilic shell. Besides, the tiny nano-size of the pluronic nanocarriers allows their accumulation within the cancer tissue through the enhanced permeability and retention (EPR) effect. Moreover, pluronics nanocarriers have been reported to overcome drug resistance by P-gp efflux system [42,43,44].

The bioenhancing effect of nanoformulated PIP (PIP-NMs) on the anticancer activity of the nanoformulated MTX (MTX-MNMs) was investigated. PIP-NMs were added to MTX-MNMs as per a certain ratio (half of PIP-NMs IC_50_) so that the PIP amount did not affect more than 25% of the cells’ population. At the two investigated time intervals, the cytotoxicity was enhanced significantly as the IC_50_ value of the PIP-NMs/MTX-MNMs mixture decreased from 3.47 to 0.627 µg/mL and from 2.099 to 0.128 µg/mL, at 24 and 48 h, respectively, as compared to MTX-MNMs.

To investigate the magnitude and phase of cell death, the mixture PIP-NMs/MTX-MNMs was selected, as it was the most powerful cytotoxic formulation. Therefore, the IC_50_ at 24 h was used to perform the flow cytometry assessment. The apoptosis/necrosis assay was performed by annexin-V/propidium iodide test to define the percent of the viable cells, early and late apoptotic cells, as well as necrotic cells after treatment with the PIP-NMs/MTX-MNMs mixture, and the results were compared to untreated cells as control. The results indicated that a limited number of the tested cells undergo necrosis, and the main tendency of cell death by the PIP-NMs/MTX-MNMs mixture was through both the early and late apoptotic stage. DNA content analysis was also performed to identify the cell cycle kinetics of MCF-7 cells after treatment with the investigated mixture; PIP-NMs/MTX-MNMs using the IC_50_ obtained at 24 h. As compared to the control, the mixture changed the cells’ population in the G0/G1 phase, which emphasized the cells’ death at the apoptosis stage. Additionally, the main cells’ population redistribution was observed at the G2/M phase due to cell cycle arrest at the G2 phase.

The Ehrlich ascites tumor is a common in vivo model and refers to undifferentiated transplantable cancer cells [56]. Due to the existence of some similarities with the human tumors as the susceptibility to the anticancer drugs and rapid growth rate [56], this model was adopted for the in vivo investigation of the treatment efficiency by MTX-MNMs before and after the fortifying by the nanoformulated bioenhancer (PIP-NMs). First, in order to determine the efficiency of the prepared nanoformulations, the tumor weight and volume were measured. The addition of PIP-NMs did not enhance the retraction of the tumor weight while the tumor volume was significantly reduced as compared to the MTX-MNMs group. Upon the assessment of the tumor markers, the addition of PIP-NMs did not significantly enhance the therapeutic effect of MTX-MNMs. Cyclin-D1 was overexpressed in 50% of breast cancer cases, so it is elicited to be one of the molecular driving forces [57,58]. Accordingly, it was screened to reveal any antagonist role mediated by the formulations. p-38 MAPK is among the protein kinases that convert the extracellular signals to cellular responses as proliferation, differentiation and transcription regulation [59]. To investigate the induced oxidative stress, SOD and MDA concentrations were analyzed. SOD is an anti-oxidant enzyme that provides protection against reactive oxygen species (ROS). It exerts its action via the catalysis of the dismutation of the of superoxide anion radicals to H_2_O_2_ [60]. The SOD level reflects the free radicals level, as it is among the protective enzymes that scavenges free radicals. Altered lipid peroxidation is one of the remarkable abnormalities in cancer tissues due to altered anti-oxidant activity. MDA is one of the end products of lipid peroxidation and is found in plentiful amounts in breast cancer [61]. AKT overexpression protects the cells from apoptosis, and thus, its level indicated induced apoptosis after treatment with the formulations [62]. The apoptosis was investigated through AKT and tunnel assay, which both confirmed the induced cell death through apoptosis by PIP-NMs/MTX-MNMs, and these results are in concordance with the in vitro apoptosis/necrosis assay. From the results of the tumor markers analysis, cyclin-D1, MDA and AKT results of MTX-MNMs were nearly the same as those of PIP-pNMs/MTX-MNMs. On the other hand, there was a significant change in the results of p-38 MAPK, SOD and the apoptosis. On the cellular level, the PIP-NMs/MTX-MNMs formula enhanced the regression of tumor via downregulation of TGF-β and Bcl-2, as confirmed by histological and immunohistochemical analysis. Although the in vivo assessment highlighted the efficient treatment of breast cancer by MTX-MNMs, the hypothesized enhancement by PIP-NMs addition was not significantly clear in all the results. These results are attributed to several probabilities as the ratio between the pluronic-loaded drugs, the cancer type and the adopted in vivo model.

## 5. Conclusions

This study aimed to fortify the MTX efficiency against breast cancer through two approaches. The first one was through mixing MTX with the bioenhancer PIP, as per a certain ratio, and the anticancer effect was confirmed upon in vitro testing on MCF-7 cells as the IC_50_ value significantly decreased. The second approach was through the nanoencapsulation of MTX and PIP individually into pluronic nanomicelles (NMs and MNMs), followed by mixing the PIP-NMs and MTX-MNMs together as per the ratio of their free drugs counterparts, and the cytotoxicity was then investigated. The nanomicelles formation was confirmed by various physical characterization techniques. From the cytotoxicity results, the pluronic encapsulation significantly enhanced the bioavailability of both MTX and PIP. Moreover, the mixing of the pluronic-encapsulated drugs significantly fortified the efficiency of the MTX as compared to either the free drug or the pluronic-encapsulated drug. The in vivo assessment was performed by adopting the Ehrlich ascites model, which confirmed the efficiency of MTX-MNMs treatment for 3 weeks, but did not confirm a significant enhancement by PIP-NMs addition. The future investigations would be directed to reveal a deeper molecular and mechanistic pathway investigations towards the optimum usage of the drug and the bioenhancer to achieve greater treatment efficiency.

## Figures and Tables

**Table 1:** Particle size and surface charge evaluation of the developed plain and drugs-loaded pluronic nanomicelles as determined by DLS.

Formulation	PS (nm)	PDI	ZP (mV)
MNMs	26.3 ± 0.1	0.43	−4.42
MTX-MNMs	104.6 ± 1.7	0.29	−3.47
NMs	96.3 ± 1.1	0.19	−19.4
PIP-NMs	114.2 ± 0.3	0.26	−8.02

**Table 2:** Cytotoxicity results of free drugs (PIP and MTX), PIP-NMs and MTX-MNMs at 24 and 48 h.

Drug μg/mL	IC_50_ Value	With 0.5 IC_50_ of Free PIP	With 0.5 IC_50_ of PIP-NMs
Free PIP (24 h)	183 ± 5.63	-	-
PIP-NMs (24 h)	11.84 ± 0.46	-	-
Free MTX (24 h)	213 ± 9.02	45.23 ± 2.91	-
Free MTX (48 h)	115.32 ± 7.08	25.84 ± 1.28	-
MTX-MNMs (24 h)	3.47 ± 0.13		0.627 ± 0.03
MTX-MNMs (48 h)	2.099 ± 0.08		0.128 ± 0.02

**Table 3:** Levels of cyclin D1 and p-38 MAPK of MTX-MNMs and PIP-NMs/MTX-MNMs -treated groups as compared to the control group. Data represented as mean ± SD (*n* = 7).

Group	Cyclin D1 (ng/mL)	p-38 MAPK (ng/mL)
Control	8.4 ± 0.21	16.6 ± 1.17
MTX-MNMs	2.2 ± 0.16	4.7 ± 0.08
PIP-NMs/MTX-MNMs	2.4 ± 0.12	3.1 ± 0.2

## Data Availability

The research data used in preparation of the manuscript will be available upon request.

## References

[1] DeSantis C.E., Bray F., Ferlay J., Lortet-Tieulent J., Anderson B.O., Jemal A. (2015). International variation in female breast cancer incidence and mortality rates. Cancer Epidemiol. Biomark. Prev..

[2] Sung H., Ferlay J., Siegel R.L., Laversanne M., Soerjomataram I., Jemal A., Bray F. (2021). Global Cancer Statistics 2020: GLOBOCAN Estimates of Incidence and Mortality Worldwide for 36 Cancers in 185 Countries. CA Cancer J. Clin..

[3] Reddy Allugunti V. (2022). Breast cancer detection based on thermographic images using machine learning and deep learning algorithms Healthcare View project Breast cancer detection based on thermographic images using machine learning and deep learning algorithms. Comput. Sci..

[4] Waks A.G., Winer E.P. (2019). Breast Cancer Treatment: A Review. J. Am. Med. Assoc..

[5] Brian P., Monahan C.J.A. (1990). Cancer Chemotherapy: Principles and Practice. Cancer Chemotherapy: Principles and Practice.

[6] Subramanian S., Kaufman B.T. (1978). Interaction of methotrexate, folates, and pyridine nucleotides with dihydrofolate reductase: Calorimetric and spectroscopic binding studies. Proc. Natl. Acad. Sci. USA.

[7] Kaasgaard T., Andresen T.L., Jensen S.S., Holte R.O., Jensen L.T., Jørgensen K. (2009). Liposomes containing alkylated methotrexate analogues for phospholipase A2 mediated tumor targeted drug delivery. Chem. Phys. Lipids.

[8] Cerqueira P., Noro J., Moura S., Guimarães D., Silva C., Cavaco-Paulo A., Loureiro A. (2019). PTS micelles for the delivery of hydrophobic methotrexate. Int. J. Pharm..

[9] Bertino J.R., Göker E., Gorlick R., Li W.W., Banerjee D. (1996). Resistance mechanisms to methotrexate in tumors. Stem Cells.

[10] Nobacci G. (2000). Neoadjuvant chemotherapy for osteosarcoma of the extremities with synchronous lung metastases: Treatment with cisplatin, adriamycin and high dose of methotrexate and ifosfamide. Oncol. Rep..

[11] Li S., Xiong Y., Zhang X. (2017). Poloxamer surface modified trimethyl chitosan nanoparticles for the effective delivery of methotrexate in osteosarcoma. Biomed. Pharmacother..

[12] Garg N.K., Singh B., Jain A., Nirbhavane P., Sharma R., Tyagi R.K., Kushwah V., Jain S., Katare O.P. (2016). Fucose decorated solid-lipid nanocarriers mediate efficient delivery of methotrexate in breast cancer therapeutics. Colloids Surf. B Biointerfaces.

[13] Matsuoka H., Ohi N., Mihara M., Suzuki H., Miyamoto K., Maruyama N., Tsuji K., Kato N., Akimoto T., Takeda Y. (1997). Antirheumatic agents: Novel methotrexate derivatives bearing a benzoxazine or benzothiazine moiety. J. Med. Chem..

[14] Nair M.G., Wang Y., Galivan J. (1993). Acquisition of Resistance to Antifolates Caused by Enhanced γ-Glutamyl Hydrolase Activity. Cancer Res..

[15] Chan E.S.L., Fernandez P., Cronstein B.N. (2007). Methotrexate in rheumatoid arthritis. Expert Rev. Clin. Immunol..

[16] Hannoodee M., Mittal M. (2022). Methotrexate. Statpearls.

[17] Yousefi G., Foroutan S.M., Zarghi A., Shafaati A. (2010). Synthesis and characterization of methotrexate polyethylene glycol esters as a drug delivery system. Chem. Pharm. Bull..

[18] Kasim N.A., Whitehouse M., Ramachandran C., Bermejo M., Lennernäs H., Hussain A.S., Junginger H.E., Stavchansky S.A., Midha K.K., Shah V.P. (2004). Molecular properties of WHO essential drugs and provisional biopharmaceutical classification. Mol. Pharm..

[19] Crivellari D., Bonetti M.C.-G. (2019). Burdens and Benefits of Adjuvant Cyclophosphamide, Methotrexate, and Fluorouracil and Tamoxifen for Elderly Patients With Breast Cancer: The International Breast Cancer Study Group Trial VII. J. Clin. Oncol..

[20] Colleoni M., Rocca A., Sandri M.T., Zorzino L., Masci G., Nolè F., Peruzzotti G., Robertson C., Orlando L., Cinieri S. (2002). Low-dose oral methotrexate and cyclophosphamide in metastatic breast cancer: Antitumor activity and correlation with vascular endothelial growth factor levels. Ann. Oncol..

[21] Poole C.J., Earl H.M., Hiller L., Dunn J.A., Bathers S., Grieve R.J., Spooner D.A., Agrawal R.K., Fernando I.N., Brunt M. (2006). Epirubicin and Cyclophosphamide, Methotrexate, and Fluorouracil as Adjuvant Therapy for Early Breast Cancer. N. Engl. J. Med..

[22] Gulfam M., Matini T., Monteiro P.F., Riva R., Collins H., Spriggs K., Howdle S.M., Jérôme C., Alexander C. (2017). Bioreducible cross-linked core polymer micelles enhance: In vitro activity of methotrexate in breast cancer cells. Biomater. Sci..

[23] Zhao L., Huo M., Liu J., Yao Z., Li D., Zhao Z., Tang J. (2013). In vitro investigation on the magnetic thermochemotherapy mediated by magnetic nanoparticles combined with methotrexate for breast cancer treatment. J. Nanosci. Nanotechnol..

[24] Chen Y., Zhang W., Gu J., Ren Q., Fan Z., Zhong W., Fang X., Sha X. (2013). Enhanced antitumor efficacy by methotrexate conjugated Pluronic mixed micelles against KBv multidrug resistant cancer. Int. J. Pharm..

[25] Hashimoto K., Yaoi T., Koshiba H., Yoshida T., Maoka T., Fujiwara Y., Yamamoto Y., Mori K. (1996). Photochemical Isomerization of Piperine, a Pungent Constituent in Pepper. Food Sci. Technol. Int. Tokyo.

[26] Elnaggar Y.S.R., Etman S.M., Abdelmonsif D.A., Abdallah O.Y. (2015). Novel piperine-loaded Tween-integrated monoolein cubosomes as brain-targeted oral nanomedicine in Alzheimer’s disease: Pharmaceutical, biological, and toxicological studies. Int. J. Nanomed..

[27] Wang-sheng C., Jie A., Jian-jun L., Lan H., Zeng-bao X., Chang-qing L. (2017). Piperine attenuates lipopolysaccharide (LPS)-induced inflammatory responses in BV2 microglia. Int. Immunopharmacol..

[28] Taqvi S.I.H., Shah A.J., Gilani A.H. (2008). Blood pressure lowering and vasomodulator effects of piperine. J. Cardiovasc. Pharmacol..

[29] Lai L.H., Fu Q.H., Liu Y., Jiang K., Guo Q.M., Chen Q.Y., Yan B., Wang Q.Q., Shen J.G. (2012). Piperine suppresses tumor growth and metastasis in vitro and in vivo in a 4T1 murine breast cancer model. Acta Pharmacol. Sin..

[30] Bhardwaj R.K., Glaeser H., Becquemont L., Klotz U., Gupta S.K., Fromm M.F. (2002). Piperine, a major constituent of black pepper, inhibits human P-glycoprotein and CYP3A4. J. Pharmacol. Exp. Ther..

[31] Volak L.P., Ghirmai S., Cashman J.R., Court M.H. (2008). Curcuminoids inhibit multiple human cytochrome P 450 (CYP). Drug Metab. Dispos..

[32] Katiyar S.S., Muntimadugu E., Rafeeqi T.A., Domb A.J., Khan W. (2016). Co-delivery of rapamycin- and piperine-loaded polymeric nanoparticles for breast cancer treatment. Drug Deliv..

[33] Kakarala M., Brenner D.E., Korkaya H., Cheng C., Tazi K., Ginestier C., Liu S., Dontu G., Wicha M.S. (2010). Targeting breast stem cells with the cancer preventive compounds curcumin and piperine. Breast Cancer Res. Treat..

[34] Schmidt B., Ferreira C., Passos C.L.A., Silva J.L., Fialho E. (2020). Resveratrol, curcumin and piperine alter human glyoxalase 1 in mcf-7 breast cancer cells. Int. J. Mol. Sci..

[35] Kumar P., Roy S., Kumar M. (2014). Pharmacokinetic study of Piperine in Wistar rats after oral and intravenous administration. Int. J. Drug Deliv..

[36] Moorthi C., Krishnan K., Manavalan R., Kathiresan K. (2012). Preparation and characterization of curcumin-piperine dual drug loaded nanoparticles. Asian Pac. J. Trop. Biomed..

[37] Shaikh J., Ankola D.D., Beniwal V., Singh D., Kumar M.N.V.R. (2009). Nanoparticle encapsulation improves oral bioavailability of curcumin by at least 9-fold when compared to curcumin administered with piperine as absorption enhancer. Eur. J. Pharm. Sci..

[38] Zhang W., Shi Y., Chen Y., Ye J., Sha X., Fang X. (2011). Multifunctional Pluronic P123/F127 mixed polymeric micelles loaded with paclitaxel for the treatment of multidrug resistant tumors. Biomaterials.

[39] Pragatheeswaran A.M., Chen S.B. (2013). Effect of chain length of PEO on the gelation and micellization of the pluronic F127 copolymer aqueous system. Langmuir.

[40] Bodratti A., Alexandridis P. (2018). Formulation of Poloxamers for Drug Delivery. J. Funct. Biomater..

[41] Valero M., Castiglione F., Mele A., Da Silva M.A., Grillo I., González-Gaitano G., Dreiss C.A. (2016). Competitive and Synergistic Interactions between Polymer Micelles, Drugs, and Cyclodextrins: The Importance of Drug Solubilization Locus. Langmuir.

[42] Duncan R., Vicent M.J. (2013). Polymer therapeutics-prospects for 21st century: The end of the beginning. Adv. Drug Deliv. Rev..

[43] Alvarez-Lorenzo C., Sosnik A., Concheiro A. (2011). PEO-PPO Block Copolymers for Passive Micellar Targeting and Overcoming Multidrug Resistance in Cancer Therapy. Curr. Drug Targets.

[44] Alakhova D.Y., Kabanov A.V. (2014). Pluronics and MDR reversal: An update. Mol. Pharm..

[45] Chen Y., Zhang W., Huang Y., Gao F., Sha X., Lou K., Fang X. (2015). The therapeutic effect of methotrexate-conjugated pluronic-based polymeric micelles on the folate receptor-rich tumors treatment. Int. J. Nanomed..

[46] Sedeky A.S., Khalil I.A., Hefnawy A., El-Sherbiny I.M. (2018). Development of core-shell nanocarrier system for augmenting piperine cytotoxic activity against human brain cancer cell line. Eur. J. Pharm. Sci..

[47] Greenshields A.L., Doucette C.D., Sutton K.M., Madera L., Annan H., Yaffe P.B., Knickle A.F., Dong Z., Hoskin D.W. (2015). Piperine inhibits the growth and motility of triple-negative breast cancer cells. Cancer Lett..

[48] Fawzi M., Mahmoud M.Y., Bakr A.F., Zaafar D., El-sherbiny I.M. (2022). Switching indication of PEGylated lipid nanocapsules-loaded with rolapitant and deferasirox against breast cancer: Enhanced in-vitro and in-vivo cytotoxicity. Life Sci..

[49] Fuliaş A., Popoiu C., Vlase G., Vlase T., Oneţiu D., Sǎvoiu G., Simu G., Pǎtruţescu C., Ilia G., Ledeţi I. (2014). Thermoanalytical and spectroscopic study on methotrexate—Active substance and tablet. Dig. J. Nanomater. Biostructures.

[50] Armagan A., Uzar E., Uz E., Yilmaz H.R., Kutluhan S., Koyuncuoglu H.R., Soyupek S., Cam H., Serel T.A. (2008). Caffeic acid phenethyl ester modulates methotrexate-induced oxidative stress in testes of rat. Hum. Exp. Toxicol..

[51] Lee A.V., Oesterreich S., Davidson N.E. (2015). MCF-7 Cells—Changing the Course of Breast Cancer Research and Care for 45 Years. J. Natl. Cancer Inst..

[52] Chiappetta D.A., Sosnik A. (2007). Poly(ethylene oxide)-poly(propylene oxide) block copolymer micelles as drug delivery agents: Improved hydrosolubility, stability and bioavailability of drugs. Eur. J. Pharm. Biopharm..

[53] Jones M.C., Leroux J.C. (1999). Polymeric micelles—A new generation of colloidal drug carriers. Eur. J. Pharm. Biopharm..

[54] Yang B., Guo C., Chen S., Junhe M., Wang J., Liang X., Zheng L., Liu H. (2006). Effect of acid on the aggregation of poly(ethylene oxide)-poly(propylene oxide)-poly(ethylene oxide) block copolymers. J. Phys. Chem. B.

[55] Bezerra D.P., de Castro F., Alves A.P.N.N., Pessoa C., de Moraes M.O., Silveira E.R., Lima M.A.S., Elmiro F.J.M., De Alencar N.M.N., Mesquito R.O. (2008). In vitro and in vivo antitumor effect of 5-FU combined with piplartine and piperine. J. Appl. Toxicol..

[56] Ozaslan M., Karagoz I.D., Kilic I.H., Guldur M.E. (2011). Ehrlich ascites carcinoma. Afr. J. Biotechnol..

[57] Arnold A., Papanikolaou A. (2005). Cyclin D1 in breast cancer pathogenesis. J. Clin. Oncol..

[58] Schuuring E. (1995). The involvement of the chromosome 11 ql 3 region in human malignancies: Cyclin D1 and EMS1 are two new candidate oncogenes—A review *. Gene.

[59] Loesch M., Chen G. (2008). The p38 MAPK stress pathway as a tumor suppressor or more?. Front. Biosci..

[60] McCord J.M., Fridovich I. (1969). Superoxide dismutase an enzymtic function for erythrocuprein (hemocuprein). J. Biol. Chem..

[61] Gönenç A., Özkan Y., Torun M., Şmşek B. (2001). Plasma malondialdehyde (MDA) levels in breast and lung cancer patients. J. Clin. Pharm. Ther..

[62] Zhou H., Li X.M., Meinkoth J., Pittman R.N. (2000). Akt regulates cell survival and apoptosis at a postmitochondrial level. J. Cell Biol..

