# Correction: Mansour et al. Dual-Enhanced Pluronic Nanoformulated Methotrexate-Based Treatment Approach for Breast Cancer: Development and Evaluation of In Vitro and In Vivo Efficiency. *Pharmaceutics* 2022, *14*, 2668

**DOI:** 10.3390/pharmaceutics15030994

**Published:** 2023-03-20

**Authors:** Amira Mansour, Mohamed Y. Mahmoud, Alaa F. Bakr, Monira G. Ghoniem, Fatima A. Adam, Ibrahim M. El-Sherbiny

**Affiliations:** 1 Nanomedicine Research Labs, Center for Materials Science, Zewail City of Science & Technology, Giza 12578, Egypt; 2 Department of Toxicology and Forensic Medicine, Faculty of Veterinary Medicine, Cairo University, Giza 12211, Egypt; 3 Department of Pathology, Faculty of Veterinary Medicine, Cairo University, Giza 12211, Egypt; 4 Department of Chemistry, College of Science, Imam Mohammad Ibn Saud Islamic University (IMSIU), Riyadh 11623, Saudi Arabia

In the original publication [1], specifically in Section 2.9. In Vivo Assessment, paragraph 1, lines 2–4, there is a need to correct some details in the ethical approval of the in vivo study.

We also highlighted the changes in Section 2.9. In Vivo Assessment, paragraph 1, lines 2–4, as well as in the Institutional Review Board Statement, with the following text.

“The in vivo experiment was performed after receiving the approval from the Institutional Animal Care and Use Committee (ARC-IACUC), Cairo, Egypt (protocol number: ARC-AH-22-29)”

“**Institutional Review Board Statement:** The in vivo experiment was performed after receiving the approval from the Institutional Animal Care and Use committee (ARC-IACUC), Cairo, Egypt (Protocol number: ARC-AH-22-29) that complies with the United Kingdom Animals Scientific Procedures Act, 1986, and the European Union directive 2010/63/EU for animal experiments, as well as the ARRIVE guidelines.”

The authors apologize for any inconvenience caused and the state that the scientific conclusions are unaffected. This correction was approved by the Academic Editor. The original publication has also been updated.
